# Single-Photon Emission Computed Tomography (SPECT-CT) as a Predictor of Pain Generators in Patients Undergoing Anterior Cervical Discectomy and Fusion (ACDF) for Axial Cervical Pain

**DOI:** 10.7759/cureus.58821

**Published:** 2024-04-23

**Authors:** Diogo Garcia, Oluwaseun O Akinduro, Gaetano De Biase, Alaa Montaser, Rodrigo Ramirez, Selby Chen, Sukhwinder Johnny S Sandhu, Kingsley Abode-Iyamah, Eric Nottmeier

**Affiliations:** 1 Neurosurgery, Mayo Clinic, Jacksonville, USA; 2 Radiology, Mayo Clinic, Jacksonville, USA

**Keywords:** ct, spect, acdf, spine, spinal surgery

## Abstract

Background: Axial neck pain is often associated with cervical instability, and surgical options are often reserved for patients with either neurological compromise or deformity of the spine. However, cervical facet arthropathy is often implicated with instability and the location of painful generators is often difficult to ascertain. Single-photon emission computed tomography (SPECT-CT) presents an adjunct to conventional imaging in the workup of patients with suspected facetogenic pain. We aimed to report our experience with patients undergoing anterior cervical discectomy and fusion (ACDF) guided by SPECT-CT for axial cervical pain.

Methods: We retrospectively identified all cases undergoing ACDF that presented with axial neck pain where correlating SPECT-CT high metabolism areas were identified. Patients were treated at a tertiary care institution between January 2018 and January 2021. Patients with positive radiotracer uptake pre-operatively were compared with patients undergoing ACDF without uptake on SPECT-CT. The pre- and post-operative patients who reported neck pain at one year were compared.

Results: Thirty-five patients were included in this retrospective cohort. The median pre- and post-intervention (at one-year follow-up) visual analog score (VAS) of patients undergoing ACDF without uptake on SPECT-CT was 7 and 3 (p<0.01), while the pre- and post-VAS for patients undergoing surgery with positive uptake on SPECT-CT was 8.5 and 0 (p<0.01). Improvement was significantly larger for patients undergoing SPECT-CT-guided ACDF (p=0.02). At one year after surgery, none of the assessed patients required additional surgical intervention.

Conclusion: This case series represents the experience of our group to date with patients undergoing SPECT-CT-guided ACDF with results suggesting potential benefit in guiding fusion.

## Introduction

Axial neck pain is common and often associated with unidentified instability (defined by Panjabi as the loss of the spine’s ability to maintain its patterns of displacement under physiologic circumstances to prevent baseline or incremental deficits, deformity, or incapacitating pain) [1]. Further, it is debilitating, with painful generators being numerous and of difficult determination [2-8]. Cervical facet joint osteoarthritis is a common etiology for instability, responsible for up to 50% of patients presenting to pain clinics with a chief complaint of neck pain [5,6]. However, conventional morphological-based imaging studies, including X-ray, computed tomography (CT), and magnetic resonance imaging (MRI), often fall short in their ability to ascertain if an osteoarthritic facet joint is truly the primary pain generator [9-14]. Further, diagnostic/treatment with intra-articular injections directed to osteoarthritic joints with local anesthetic and steroids has limited available data supporting its efficacy in predicting long-term response to surgical arthrodesis of the affected joint [15,16]. More recently, functional imaging methods have been used to locate the source(s) of pain. Such methods include bone scintigraphy with technetium 99m-methylene diphosphonate (MDP). It has been suggested to depict osteoblastic activity and synovial changes, which can be seen in patients with inflammation or hyperemia [17]. Single-photon emission computed tomography (SPECT) allows for three-dimensional (3D) image acquisition of typical two-dimensional (2D) planar data. Hybrid SPECT-CT is a SPECT scan fused with a corresponding CT scan [17-19]. However, it remains controversial whether positive uptake on a radionucleotide study is a predictor of favorable response to surgical arthrodesis, with only a small case series being available in the literature and no prior work evaluating an anterior approach for surgical arthrodesis of the cervical spine [4,8]. Here, we aimed to report our experience using SPECT-CT before anterior cervical discectomy and fusion (ACDF) and to evaluate the potential correlation between pre-operative positive uptake and patient-reported outcomes after decompression and fusion.

## Materials and methods

Patient selection

We performed a single-institution, retrospective analysis of prospectively collected data evaluating patient-reported outcomes in patients undergoing ACDF for chronic axial neck pain with prior SPECT-CT between January 2018 and January 2021. Patients with positive radionuclide uptake in all fused segments were compared with patients undergoing ACDF and negative uptake on SPECT-CT. This study was reviewed and approved by the Institutional Review Board (18-003951). Patients were followed for a minimum of one year after intervention.

Inclusion and exclusion criteria

The inclusion criteria for this study involve patients with a clinical diagnosis of cervical axial neck pain persisting for at least six months and not showing improvement with medical management. Exclusion criteria encompass known causes of neck pain such as trauma, infection, tumor, and iatrogenic complications. Additionally, patients with isolated radiculopathy or myelopathy only are excluded, along with those having a follow-up time of less than one year.

SPECT-CT protocol

Radionucleotide imaging study in our institution is reserved for pre-operative or pre-procedural evaluation of patients with inconclusive clinical-radiographic findings and persistent pain. Our SPECT-CT protocol has further been validated in a large study evaluating SPECT-CT to guide targeted facet injections in patients with back pain [4].

Patient outcomes

From each patient, pre-operative, surgical, and post-operative data was collected. This included the number of levels being operated on, pre-operative comorbidities, intra-operative complications, blood loss, and post-operative improvement. Baseline patient-reported outcomes including cervical pain were included at the time of surgical consultation and at one-year follow-up.

Statistical analysis

Categorical variables were described using absolute and relative frequencies, while continuous variables were described using medians and interquartile ranges. Pre-intervention pain scores of patients undergoing ACDF were compared with post-intervention pain scores by means of a Mann-Whitney U test. Statistical significance was considered to be a p-value <0.05.

## Results

Patient population and clinical presentation

A total of 46 patients presented with cervical spondylosis and a pre-operative SPECT-CT before ACDF was eligible for screening. Of the 46, 11 patients were excluded due to lack of appropriate follow-up, and the remaining 35 were enrolled in the study. Of these 35, 15 underwent uptake-targeted ACDF (u-ACDF) (nine male and six female). Patients undergoing u-ACDF did not differ from patients undergoing non-uptake-targeted ACDF (nu-ACDF) in age at the time of surgery (p=0.285), average BMI (p=0.904), number of comorbidities (p=0.099), average blood loss (p=0.549), number of intra-operative (p=0.999) and post-operative (p=0.429) complications. A complete description of the patient’s demographic information is included in Table 1.

**Table 1:** Demographic and pre-morbid characteristics of included patients BMI: body mass index; COPD: chronic obstructive pulmonary disease; DVT: deep venous thrombosis; IQR: interquartile range; OSA: obstructive sleep apnea; PE: pulmonary embolism; TIA: transient ischemic attack; u-ACDF: uptake-targeted ACDF; nu-ACDF: non-uptake-targeted ACDF ^a^ Persistent dysphagia

	All patients (n=35)	u-ACDF (15)	nu-ACDF (n=20)	p-value
Age at surgery (years) - median (IQR)	57 (11)	54 (10)	60.5 (12)	0.285
Gender (male) - n (%)	19 (54)	9 (60)	10 (50)	0.734
Caucasian ethnicity - n (%)	27 (77)	11 (73)	16 (80)	0.700
One level - n (%)	22 (63)	10 (67)	12 (60)	0.737
Two levels - n (%)	7 (20)	3 (20)	4 (20)	0.999
Three levels - n (%)	4 (11)	3 (20)	1 (5)	0.292
Four levels - n (%)	2 (6)	1 (7)	1 (5)	0.999
Number of comorbidities - median (IQR)	3 (2)	4 (3)	3 (1.5)	0.099
BMI (kg/m^2^) - median (IQR)	30.1 (7)	30.1 (4.2)	30 (7.5)	0.904
Hypertension - n (%)	11 (31)	4 (27)	7 (35)	0.721
Diabetes - n (%)	11 (31)	5 (33)	6 (30)	0.999
Hyperlipidaemia - n (%)	15 (43)	6 (40)	9 (45)	0.999
Coagulopathy - n (%)	0	0	0	0.999
Coronary artery disease - n (%)	2 (6)	1 (7)	1 (5)	0.999
Heart failure - n (%)	0	0	0	0.999
Chronic kidney disease - n (%)	0	0	0	0.999
Stroke - n (%)	0	0	0	0.999
TIA - n (%)	1 (3)	0	1 (5)	0.999
Arrhythmia - n (%)	2 (6)	1 (7)	1 (5)	0.999
DVT - n (%)	3 (9)	1 (7)	2 (10)	0.999
PE - n (%)	1 (3)	0	1 (5)	0.999
COPD - n (%)	3 (9)	1 (7)	2 (10)	0.999
Asthma - n (%)	1 (3)	0	1 (5)	0.999
OSA - n (%)	4 (11)	2 (13)	2 (10)	0.999
Blood loss - median (IQR)	130 (150)	150 (150)	115 (125)	0.549
Intra-operative complications - n (%)	0	0	0	0.999
Post-operative complications - n (%)	1 (3)	1^a ^(7)	0	0.429

Management and patient-reported outcomes

**Figure 1:**
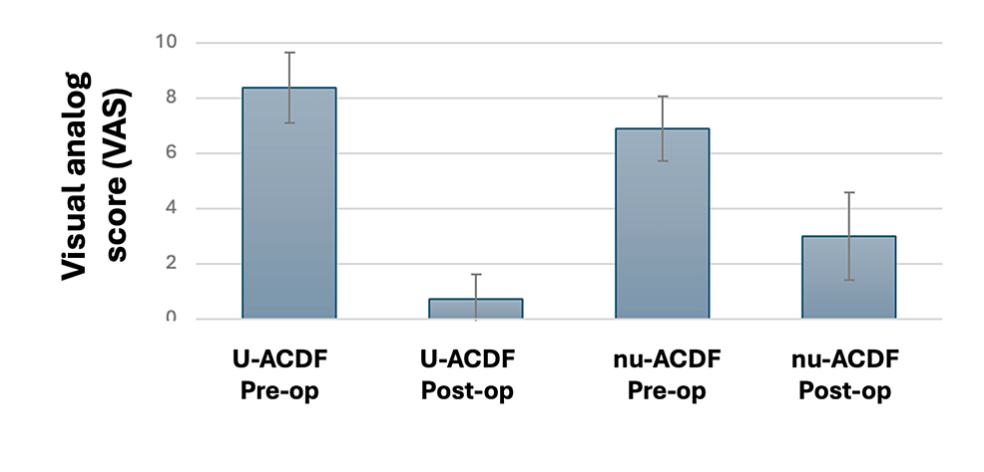
Pre- and post-operative visual analog scores (VAS) for u-ACDF and nu-ACDF u-ACDF: uptake-targeted ACDF; nu-ACDF: non-uptake-targeted ACDF

Twenty-two of the assessed patients underwent a one-level ACDF (22/35), with the most common spinal level being C6-7. Another seven (7/35), four (4/35), and two (2/35) underwent a two-, three-, and four-level ACDF, respectively. In patients undergoing u-ACDF, all levels with positive uptake were targeted. The median length of stay was one night, with surgical complications being limited to one case of persistent dysphagia after surgery. A complete description of the included patients is available in Figure 1.

The median pre- and post-intervention (at one-year follow-up) visual analog scores (VAS) of patients undergoing nu-ACDF were 7 and 3 (p<0.01), while the pre- and post-VAS for patients undergoing surgery after u-ACDF was 8.5 and 0 (p<0.01). Improvement was significantly larger for patients undergoing u-ACDF (p=0.02). At one year after surgery, none of the assessed patients required additional surgical intervention.

## Discussion

In this study, we report our experience with patients undergoing ACDF after SPECT-CT, when prior imaging findings were inconclusive. In our experience, patients undergoing ACDF for levels targeted at positive uptake were found to have good surgical outcomes after one year of follow-up. While patients undergoing ACDF for levels with negative or mixed SPECT-CT also saw improvements in their pain scores, VAS pain changes were significantly larger for patients with uptake on pre-operative SPECT-CT. This is relevant, as it corresponds to one of the first (and the largest) reports illustrating the potential benefit of incorporating SPECT-CT in the pre-operative evaluation of patients undergoing ACDF for cervical arthropathy with inconclusive findings on traditional imaging modalities.

Our results are in line with prior work, with Tender et al. suggesting the presence of abnormal radiotracer uptake on SPECT-CT as an accurate predictor for the need for potential instrumented fusion, with a significant drop in self-reported VAS six months after surgery (both from anterior and posterior approaches) in patients with pre-operative localized foci of abnormal spinal radiotracer uptake [7]. Other studies from our group [14] and others [18-20] had also previously suggested the potential benefit of incorporating SPECT-CT to guide targeted injections for facetogenic pain, with results suggesting that SPECT-CT in selected patients can help guide injections to painful generators. However, the role of SPECT-CT in guiding the placement of instrumentation remains controversial with no systematic guidelines recommending its incorporation in clinical workflows at this time. Our report represents the experience of a large tertiary academic center with good surgical outcomes where SPECT-CT was used to guide the extent of fusion in patients with chronic axial neck pain with inconclusive imaging findings on CT and MRI but with positive uptake on pre-operative SPECT-CT.

This study has important limitations. This is a retrospective single-institution study at a large tertiary care academic institution, which might limit its external validity to other healthcare settings. However, it represents a real clinical experience with long-term follow-up of patients commonly seen in community settings. The sample size is relatively small, which might have affected the precision of our estimates and observations. However, the aim of our study was to report the experience of our center with pre-operative SPECT-CT in patients with inconclusive findings, which we expect to lay the foundation for future randomized studies with control groups. However, it also has important strengths. The data collection, including patient-reported neck pain pre- and post-procedure, was independently collected by personnel not involved in this study. A potential limitation arises from the fact that patient-reported neck pain before surgery was collected through phone calls. However, this is a commonly used method in observational studies on this topic [7,20]. To minimize potential information bias, professionals collecting patient-reported outcome data are trained to do so and are blinded to whether patients were submitted to an SPECT-CT before the procedure. In fact, this study provides results from a real clinical scenario in which the real benefit of employing SPECT-CT in a tertiary academic center was evaluated. Further, the short follow-up time could have also limited our observations.

## Conclusions

In conclusion, we present the experience of our center using SPECT-CT to guide the extent of fusion in patients with axial neck pain and inconclusive findings on CT and MRI, with good surgical outcomes. Future studies are needed to be aimed at establishing clear guidelines before the generalized incorporation of SPECT-CT in clinical workflows.
